# Functional Microbial Communities in Hybrid Linear Flow Channel Reactors for Desulfurization of Tannery Effluent

**DOI:** 10.3390/microorganisms10112305

**Published:** 2022-11-21

**Authors:** Emma J. Horn, Rob P. van Hille, Oluwaseun O. Oyekola, Pamela J. Welz

**Affiliations:** 1 Applied Microbial and Health Biotechnology Institute, Cape Peninsula University of Technology, Bellville, Symphony Way, Bellville, Cape Town 7530, South Africa; 2 Department of Chemical Engineering, Cape Peninsula University of Technology, Bellville, Symphony Way, Bellville, Cape Town 7530, South Africa; 3 The Moss Group, 24 Cornuta Avenue, Tokai, Cape Town 7945, South Africa

**Keywords:** dissimilatory sulfite reductase, elemental sulfur, sulfate reduction, sulfide oxidation, methanogenesis, Petrimonas, Dethiosulfovibrio

## Abstract

Recent research has demonstrated that hybrid linear flow channel reactors (HLFCRs) can desulfurize tannery effluent via sulfate reduction and concurrent oxidation of sulfide to elemental sulfur. The reactors can be used to pre-treat tannery effluent to improve the efficiency of downstream anaerobic digestion and recover sulfur. This study was conducted to gain insight into the bacterial communities in HLFCRs operated in series and identify structure-function relationships. This was accomplished by interpreting the results obtained from amplicon sequencing of the 16S rRNA gene and quantification of the dissimilatory sulfite reducing (*dsrB*) gene. In an effort to provide a suitable inoculum, microbial consortia were harvested from saline estuaries and enriched. However, it was found that bioaugmentation was not necessary because native communities from tannery wastewater were selected over exogenous communities from the enriched consortia. Overall, *Dethiosulfovibrio* sp. and *Petrimonas* sp. were strongly selected (maximum relative abundances of 29% and 26%, respectively), while *Desulfobacterium autotrophicum* (57%), and *Desulfobacter halotolerans* (27%) dominated the sulfate reducing bacteria. The presence of elemental sulfur reducing genera such as *Dethiosulfovibrio* and *Petrimonas* is not desirable in HLFCRs, and strategies to counter their selection need to be considered to ensure efficiency of these systems for pre-treatment of tannery effluent.

## 1. Introduction

During the tanning process, the beamhouse and tanyard operations generate saline wastewaters with high concentrations of total dissolved solids and organics, typically measured as chemical oxygen demand (COD) and/or biological oxygen demand (BOD) [1]. Beamhouse effluents also contain high concentrations of ammonia/ammonium (NH_3_/NH_4_^+^) and sulfides (HS^−^), while conventional (wet-blue) tanyard effluents contain a range of contaminants, including dyes, chromium (Cr) and sulfates (SO_4_^2−^) [2]. 

The organic and SO_4_^2−^ rich effluent has potential for concurrent biogas generation and wastewater remediation via anaerobic digestion (AD) and it has been shown that saline inhibition of AD may be overcome by acclimation of the functional microbial consortia to increasing concentrations of sodium chloride (NaCl) [3]. However, the methanogenic archaea (methanogens) are pH sensitive, and are inhibited by high concentrations of sulfur species (S), particularly HS^−^ [4]. On the other hand, the sulfidogenic sulfate-reducing bacteria (SRB) can be acidophilic and/or neutrophilic and/or alkaliphilic, allowing the species to collectively thrive in a range of habitats [5]. During AD, the methanogens and SRB compete for electron donors and COD to SO_4_^2−^ ratios >10.0 generally encourage methanogenesis over sulfidogenesis provided the retention time, pH and concentrations of potential inhibitors are also favourable [3]. Therefore, to reduce the competition between methanogens and SRB and lower the concentration of potentially toxic sulfur species the tannery effluent should be pre-treated prior to AD.

During conventional treatment of tannery effluent, sulfide is commonly dealt with during primary treatment, where suspended solids and metals are also removed [2]. Active physicochemical treatments such as stripping (H_2_S gas), precipitation or oxidation are effective at removing HS^−^. However, drawbacks include the high energy, capital and chemical costs, as well as long reaction times and the generation of large volumes of sludge that require disposal [1,6]. These technologies also fail to address the primary S species in the effluent, SO_4_^2−^, which can be converted to HS^−^ under anaerobic conditions. 

Biological methods for partial sulfide oxidation (SO) of HS^−^ to elemental sulphur (S^0^) have been investigated for removal of S from a range on industrial effluents. Aerobic/chemotrophic and anaerobic/phototrophic bioreactors have been shown to be highly cost effective and capable of high removal efficiencies while producing minimal sludge for disposal at laboratory scale [7]. However, few researchers have focused on tannery effluent due to its complex nature and high salinity. 

Similarly, the removal of SO_4_^2−^ from tannery wastewater (TWW) has received limited attention. In an up-flow anaerobic sludge bed reactor, completely stirred tank reactor and trench reactor, a group of researchers [8] were all able to achieve 60–80% SO_4_^2−^ removal from a TWW feed with a SO_4_^2−^ concentration of 1800 mg/L. Others inoculated an upflow anaerobic sequencing batch reactor with *Citrobacter freundii* CZ1001 and reached a SO_4_^2−^ removal efficiency of 90% from tannery effluent with a SO_4_^2−^ loading of 1069 mg/L [9]. These studies focused on performance, rather than developing an understanding of microbial community structure, succession and possible relationships between community structure and performance.

The most common genera found in bioreactors treating SO_4_^2−^ rich effluents are the incomplete organic oxidising genera *Desulfovibrio*, *Desulfobulbus*, *Desulfomicrobium* and the complete organic oxidizer, *Desulfobacter* [10]. Apart from the variety of carbon sources that SRBs can utilise as electron donors, reduction of other oxidized electron acceptors such as sulfite (SO_3_^2−^) and thiosulphate (S_2_O_3_^2−^) is also common, as well as nitrate (NO_3_^−^) and nitrite (NO_2_^−^) for some [10].

The novel hybrid linear flow channel reactor (HLFCR) is a semi-passive system that allows sulfate reduction (SR) and partial SO of HS^−^ to S^0^ to occur concurrently [11]. The principle of this single reactor system is based on the unique hydrodynamics that supports biological SR in the anaerobic bulk volume and partial SO in a floating sulfur biofilm (FSB) that forms at the air-liquid interface. The FSB limits O_2_ mass transfer and creates a microaerobic environment within the biofilm where the pH and redox conditions are suitable for partial SO [11]. At laboratory scale, HLFCRs have been shown to remove up to 97% of S from synthetic mine-impacted water by conversion of S species to S^0^ that can then be harvested as a value-added product from the FSB [11]. 

It was hypothesized that HLFCRs may be suitable for pre-treatment of TWW to reduce the concentration of S species to non-inhibitory concentrations while preserving sufficient organics for downstream AD using anaerobic sequencing batch reactors [12]. To this end, microbial consortia were harvested from saline estuaries in South Africa and enriched for SR and SO in a laboratory setting and subsequently used to inoculate HLCFRs for pre-treatment of TWW. Operational performance results validated the hypothesis, although biofilm formation was not as efficient as in previous studies [11,13] using synthetic wastewater. Detailed performance results have been published elsewhere [14].

The relationship between microbial community structure and process performance is critical, particularly in complex matrices such as tannery effluent. This is the first time that microbial communities in HLFCRs treating TWW have been investigated. The main focus of this study was to evaluate the SRB community compositions and succession in the HLCFRs using molecular tools based on amplification of a ~350 base-pair (bp) fragment of the β-subunit of the *dsrB* gene as a phylogenetic marker. To gain insight into the contribution of other bacterial taxa, including SO species, amplicon sequencing of a fragment of the 16S rRNA gene was also performed on selected samples. 

## 2. Materials and Methods

### 2.1. Tannery Wastewater and Inoculum

Five batches of both raw and partially treated TWW (Table 1) were obtained over a 3-month period from a large tannery in South Africa that processes approximately 100,000 salted bovine hides and 250,000 ovine skins each month. The effluent was designated according to the concentration of total organic carbon (TOC): raw TWW as high TOC (H-TOC) and partially treated TWW as low TOC (L-TOC). Copper (Cu), Cobalt (Co), cadmium (Cd), nickel (Ni). Lead (Pb) and aluminium (Al) were all present in concentrations < 0.1 mg/L (data not shown). The H-TOC and L-TOC effluents were blended to obtain similar concentrations of SO_4_^2−^ for the experimental reactor feed. Details about the biological TWW treatment system used to obtain the partially treated effluent cannot be provided for reasons of confidentiality. 

Five consortia (consortia A–E) were cultured from samples sourced from various saline estuaries in South Africa (sites A–E). In order to select for saline-adapted SR and SO microbial species, the samples were inoculated into lactate-supplemented artificial seawater (23 g/L NaCl, 4.01 g/L sodium sulfate (Na_2_SO_4_), 0.67 g/L potassium chloride (KCl), 0.2 g/L sodium hydrogen carbonate (NaHCO_3_), 0.03 g/L boric acid (H_3_BO_4_) and cultured in increasing volumes over 39 days. Thereafter, the consortia were assessed for their SO and SR ability (data not shown), and the two most promising candidates (designated A and B) were chosen. The characteristics of the estuarine water at sites A and B were: pH 7.3 and 6.6; conductivity 43 and 49 mS/cm; HS^−^ 0.59 and 0.30 mg/L, and SO_4_^2−^ 2295 and 2300 mg/L, respectively. After selection, consortia A and B were adapted to TWW (batch 2) for 39 days to form the final inoculum (inoculum AB).

### 2.2. Set-Up and Operation, and Sampling Regime of Hybrid Linear Flow Channel Reactors

**Figure 1:**
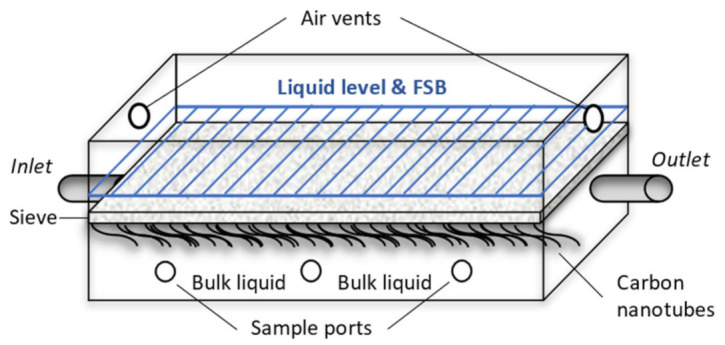
Simplified diagram of a hybrid linear flow channel reactor.

The HLFRs are described in detail elsewhere [14]. Briefly, each had a working volume of 2.1 L and were fitted with a harvesting screen for FSB recover just below the air-liquid interface. A strip of carbon microfibres were positioned in the bulk volume to provide attachment sites for bacteria to encourage biomass retention and prevent washout during continuous operation. Samples were taken at three different sampling points between the inlet and outlet (Figure 1). 

**Figure 2:**
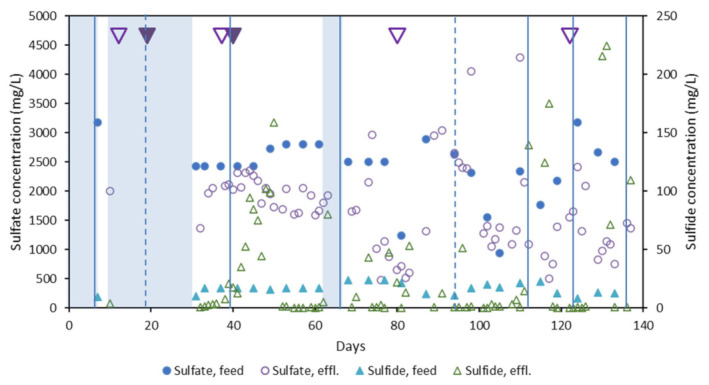
Change in the sulfide and sulfate concentrations over the HLFCR system adapted from [14]. Dashed and full vertical lines indicate biofilm harvest and disruption events, respectively, and shading indicates periods of batch operation. Effl. = effluent.

Initially, HLFCR1 was filled with 100% TWW, while HLCFR2 was filled with a mixture of inoculum AB culture and TWW (50% *v*/*v*). During start-up, HLFCR1 was operated in batch and continuous modes intermittently as previously described [14] until stable operation was achieved in continuous mode with 4-day hydraulic retention time (HRT), without recycle, after 33 days. Start-up of HLFCR2 in batch mode took place after HLFCR1 had been operational for 42 days. On day 68 of the experimental period, the reactors were connected in series and operated at 4-day HRT, without recycle until the end of the experimental period (140 days in total). Samples of effluent and bulk liquid were taken daily using sterile syringes and needles from the sample ports and outlet, respectively (Figure 1) The biofilm was physically disrupted and harvested from the screen periodically. The harvesting periods are depicted in Figure 2. 

### 2.3. Physicochemical Analyses

Liquid samples were drawn from the bulk liquid using a syringe and hypodermic needle through the sampling ports. The hydrogen sulfide (HS^−^), pH and redox measurements were performed immediately. Thereafter, the samples were centrifuged and the sulfate (SO_4_^2−^) and soluble COD concentrations were determined on filtered (0.45 μm) supernatant fluid. The HS^−^ and SO_4_^2−^ concentrations were determined using N,N-dimethyl-p-phenylenediamine (DMPD) and barium chloride (BaCl) techniques, respectively [15]. The COD was determined using Merck Spectroquant^®^ (Darmstadt, Germany) test reagents and kits according to the manufacturers’ instructions as previously described [14]. 

### 2.4. Microbial Analyses

#### 2.4.1. Extraction of Deoxyribonucleic Acid

Total genomic DNA was extracted from centrifuged pellets of the enriched consortia cultures (16 mL), bulk liquid of the HLFCRs (16 mL) and TWW (4 mL) as well as the biofilm (0.25 g FSB) samples using Qiagen (Hilden, Germany) PowerLyzer Ultraclean DNA extraction kits and Powerlyzer PowerSoil DNA isolation kits for the liquid and solid samples, respectively, according to the manufacturers’ instructions. Each of the extractions were performed in duplicate and the DNA concentrations were measured using a Jenway Genova (Bibby Scientific, Staffordshire, UK) NanoDrop spectrophotometer. Equimolar amounts of each duplicate were combined for molecular studies. 

#### 2.4.2. Amplicon Sequencing

Amplicon sequencing was performed at Molecular Research laboratories (MR DNA) (Shallowater, TX, USA) according to their established in-house protocols. Briefly, metagenomic DNA was used to amplify: (i) the V4 region of the small subunit (SSU) of the 16S rRNA gene using the primer pairs 515F-Y [16] and revised 806-R [17] and (ii) a ~350 base-pair (bp) fragment of the β-subunit of the dissimilatory sulfite reductase gene (*dsrB*) using the primer pairs dsr2061F [18] and dsr4R [19], with the forward primers being barcoded for both amplifications. 

Initial denaturation steps at 95 °C for 5 min were followed by 30 cycles of denaturation (95 °C for 30 s), annealing (53 °C for 40 s) and extension (72 °C for 1 min) and final elongation (72 °C for 10 min) using the Qiagen HotStarTaq Plus Master Mix kit. The PCR products were quality checked by visualisation in 2% agarose gel. Aliquots of samples were multiplexed using unique dual indices. Based on their DNA concentrations and molecular weights, samples were pooled in equal ratios, purified using calibrated Ampure XP beads (Beckman Coulter, Brea, CA, USA) and used to prepare the DNA library according to the Illumina TruSeq protocol. 

Sequencing was performed using an Illumina MiSeq instrument according to the manufacturer’s instructions. The data was analysed using the MR DNA analysis pipeline. The sequences were joined together, the barcodes were removed, and those with <150 bp and/or ambiguous base calls were removed. Quality filtration was performed by applying a maximum expected error threshold of 1.0, after which the sequences were dereplicated and denoised by removing unique sequences identified by PCR error points and chimeras in order to generate zero radius operational taxonomic units (zOTUs). The zOTUs were assigned taxonomic classification using BLASTn against a curated database derived from the Ribosomal Database Project II (RDP II) and the National Centre for Biotechnology Information (NCBI) databases (http://rdp.cme.mus.edu and http://www.ncbi.nlm.nih.gov accessed on 15 August 2022). Rarefaction is a widely applied method of normalizing the sequencing output data by randomly discarding reads so that all the samples contain the same number of reads as the sample with the lowest number of reads [20,21,22]. The data was not rarefied in this study because it has been well argued that this practice discards vast amounts of potentially important information [23] and introduces significant negative bias [20].

#### 2.4.3. Quantitative Polymerase Chain Reaction

Copy numbers of the *dsrB* gene fragment were determined as previously described [24] using the same primer pair as per the amplicon sequencing and *dsrB* amplicons cloned into plasmids to construct a standard curve. 

#### 2.4.4. Statistical Analyses

All statistical analyses were conducted using Primer 7^®^ software (Primer-e, Quest Research Limited, Auckland, New Zealand). The relative abundances (RA) of the zOTUs obtained from analysis of *dsrB* and 16S rRNA amplicon sequencing were used to calculate univariate diversity indices. Various multivariate statistical analyses were also performed. Data was square root transformed and used to construct Bray–Curtis similarity plots. The similarity plots were used for: (i) one-way unordered analysis of similarity (ANOSIM), (ii) cluster analyses (group average linkages), and (iii) non-metric multidimensional scaling (nMDS). Results were either tabulated (ANOSIM) or used to construct nMDS plots overlayed with the results of the cluster analyses.

Fourth root transformed and normalised physicochemical (abiotic) data was used to construct similarity matrices based on Euclidian distances. The data was then analysed using principal component analyses (PCA) and one-way unordered ANOSIM. 

To determine which abiotic parameters were the most significant drivers of microbial community selection, BEST analyses of Spearman rank correlations were performed on the afore-mentioned Bray–Curtis (biotic) and Euclidian distance (abiotic) similarity matrices. If significant, the ‘best’ correlated parameters were used to construct LINKTREE plots. 

Significance levels for all data is defined as: <0.05 * ≥ 0.01 > ** 0.005 ≥ *** throughout the manuscript unless otherwise stated. 

## 3. Results and Discussion

Pre-treatment of TWW for removal and recovery of S species using HLFCRs has been conceptually proven and detailed performance results have been published elsewhere [14]. It was conclusively demonstrated that pre-treatment of TWW in HLFCRs renders it more amendable to AD, most notably due to the reduction in the concentrations of toxic HS^−^ species. Briefly, HLFCR1 reached a steady-state during continuous operation on day 40 of the 140-day experimental period, with an average SO_4_^2−^ reduction rate (SRR) of 444 mg/L.day^−1^. Pseudo-steady state, specifically with respect to sulfide in the effluent, was maintained for the remaining 100 days, with only minor disruption due to effluent port blockages. Higher SRR were achieved in HLFCR1, the first reactor in series (up to 1049 mg/L.day^−1^) than in HLFCR2 (up to 513 mg/L.day^−1^). The higher rate in HLFCR1 was attributed to the presence of higher concentrations of SO_4_^2−^ and more readily biodegradable carbon substrates (soluble COD). Near complete removal of HS^−^ was achieved, with final effluent values of <5 mg/L when the flow out of the reactor was unrestricted (Figure 2). Occasional blocking of the effluent port by elemental sulfur resulted in a rise in liquid height and meant that some effluent samples were effectively bulk liquid, accounting for the high effluent sulfide values. A complete FSB was formed with a S content of 38–76% (*n* = 4), demonstrating the potential of the reactors for S recovery [14]. Biofilm formation was slower than previously reported and the S content was also lower [11,13]. This resulted in more SO occurring below the biofilm with colloidal S visible in the reactor. The frequency of biofilm disruption and harvesting is determined by the rate of formation and thickening of the biofilm. Beyond a threshold thickness, the rate of O_2_ ingress is restricted to the point where sulfide oxidation within the biofilm becomes limiting, resulting in steady increase in effluent sulfide concentration.

This is the first time that microbial communities in HLFCRs treating TWW have been investigated. The main focus of this study was to characterize the environmental enrichments and endogenous TWW populations and evaluate the SRB and SO community compositions and succession in the HLCFRs using molecular tools as outlined in the graphical abstract. It was hypothesized that the enriched consortia obtained from saline estuaries would contain SRB and SO species able to proliferate in the saline TWW (Table 1).

### 3.1. Influence of Endogenous and Exogenous Bacterial Communities on Bacterial Community Composition in Hybrid Linear Flow Channel Reactor Pre-Treating Tannery Wastewater 

Overall, the SRB communities in the enriched consortia were significantly different from the SRB communities in the HLFCRs and the batches of TWW (*viz*. all the other samples, Table 2). 

**Figure 3:**
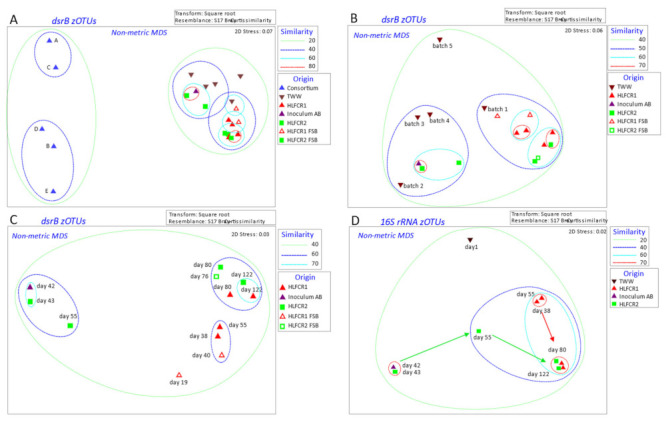
nMDS plots with data points representing Bray–Curtis similarities of all transformed: *dsrB* amplicon zOTU samples (**A**), *dsrB* amplicon zOTU samples with consortia removed (**B**), *dsrB* amplicon zOTU samples with consortia and tannery effluent removed (**C**), and all 16S rRNA amplicon zOTU samples (**D**). Samples of TWW batches 1–5 taken on day 12, 33, 47, 72, 96, respectively.

The ANOSIM results were validated by visualization of the Bray–Curtis similarity in nMDS plots, where data points denoting the SRB community structures in the enriched consortia (A–E) grouped well away from those of the TWW, bulk liquid and FSB of both HLFCRs (Figure 3A). 

The SRB community compositions in Inoculum AB also differed from those in the enriched consortia (Figure 3B,C). In contrast to HLFCR1, which relied on the endogenous communities in the TWW, HLFCR2 had been inoculated with Inoculum AB during start up. Inoculum AB was pre-acclimated in 50% (*v*/*v*) batch 2 TWW, and the results indicate that the SRB community in HLFCR2 was dominated by endogenous zOTUs from the, TWW, not from the enriched consortia A or B, because (i) data points denoting the SRB community compositions in the initial samples from HLFCR2, batch 2 TWW and Inoculum AB grouped closely together, and (ii) data points denoting the SRB community compositions in Inoculum AB grouped away from those denoting enriched consortia A and B. This suggests that there is no need for bioaugmentation of HLFCRs treating TWW from the study facility.

The data points representing the SRB in different batches of TWW grouped away from one another in the nMDS plots, but the data point representing batch 1 (day 1–33) grouped closely with the data points representing all the samples from HLFCR1, as well as HLFCR2 towards the end of the experiment (day 80–122), sharing 50% similarity (Figure 3B). These results indicated that overall, the SRB communities in TWW batch 1 were most suited to the physicochemical milieu in the HLFCRs. 

The most noteworthy finding was that the SRB communities in both HLFCRs stabilized from day 80 to the end of the microbial study (day 122) and were highly similar to the original communities in HLFCR1 (Figure 3C), indicating that the endogenous SRB communities in batch 1 of TWW were more resilient within the systems than the exogenous communities or endogenous communities in the other batches of TWW. According to ANOSIM, the TWW communities were significantly different to those in HLFCR2, but not HLFCR 1 (Table 2), which seems counter-intuitive. However, the ANOSIM analysis combined all the TWW samples to provide an overall picture and did not account for the influence of individual TWW batches. In a full-scale 2-stage aerobic TWW treatment system in China bioaugmented with a microbial consortium consisting of 13 genera including 33.1% *Gluconobacter*, 32.8% *Acetobacter* and 26.3% *Lactobacillus*, an average COD removal efficiency of 95.2% was achieved [25]. Results were not compared with a non-augmented system, so the positive effect of bioaugmentation was not statistically validated. However, members of the consortia harbored numerous functional gene clusters capable of degrading a range of organic compounds. 

Changes in the overall bacterial community composition were determined by evaluating the results of 16S rRNA amplicon sequencing. The results mimicked those found with the SRBs in that the original communities in HLFCR2 were highly similar to those in Inoculum AB and TWW batch 2, and the communities in both HLFCRs evolved initially and then stabilized so that the communities in both HLFCRs were >70% similar between day 80 and 122 of the study (Figure 3D). 

### 3.2. Influence of Physicochemical Parameters on Community Selection

**Figure 4:**
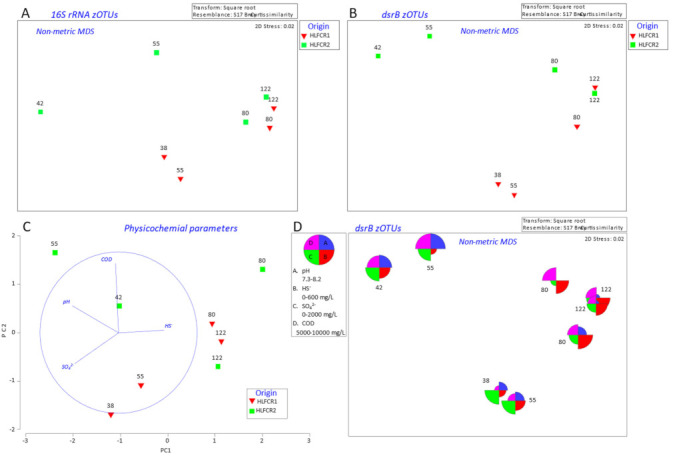
nMDS plots with data points representing Bray–Curtis similarities of transformed: 16S rRNA amplicon zOTU samples (**A**) and *dsrB* amplicon zOTU samples (**B**). PCA plot of Euclidean distance similarity of transformed physicochemical data (**C**), and bubble overlays of physicochemical data on plot B (**D**).

To evaluate the effects of physicochemical parameters on the overall bacterial and SRB community compositions, the spatial distribution patterns determined from the biotic and abiotic data were compared using nMDS plots of the Bray–Curtis similarity results obtained from 16S rRNA amplicon sequencing (Figure 4A) and *dsrB* amplicon sequencing (Figure 4B), and a PCA plot of the Euclidean distance similarity of the physicochemical data (Figure 4C), with all plots being aligned with one another. By examining the plots, it was clear that the physicochemical profiles and the microbial community compositions within samples were similar and stable from day 80 to day 122 of the study. In general, the COD concentration was higher and the pH lower during this period (Figure 4D). 

No significant overall correlation was found between the bacterial community composition and the abiotic data (BEST Global R = 0.595; *p* > 5) for the range of parameters analyzed, although the highest R value was obtained for pH (0.595) and combined pH and HS^−^ concentration (0.518). In contrast, a significant overall correlation was found between the SRB community structures and the abiotic data (BEST Global R = 0.779 **). This was attributed to the fact that the parameters that were measured were likely to have a significant influence on the SRB communities, but not necessarily the non-SRB communities. 

**Figure 5:**
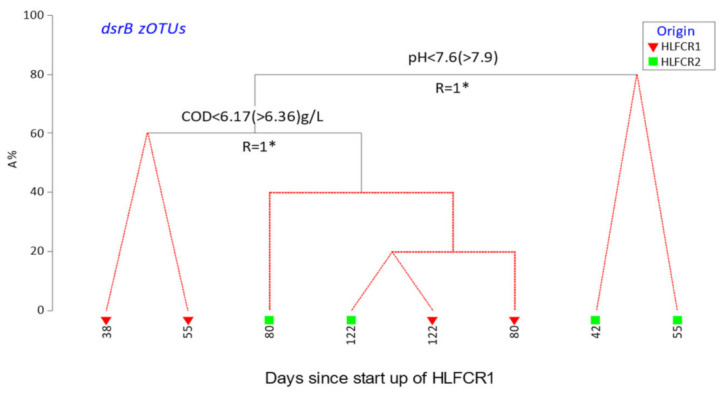
LINKTREE plot of clustering of sulfite reducing bacterial communities related to physicochemical data. * significant (*p* < 0.05).

The binary divisive cluster plot (LINKTREE) substantiated the results obtained with the nMDS and PCA plots—that the primary drivers of SRB community structures were pH and COD concentration. In this instance, selection of the final (day 80 and day 122) communities in both HLFCRs was firstly by pH (<7.6) and then COD (>3.36 g/L) (Figure 5).

### 3.3. Selection of Bacterial Taxa in Hybrid Linear Flow Channel Reactors

**Figure 6:**
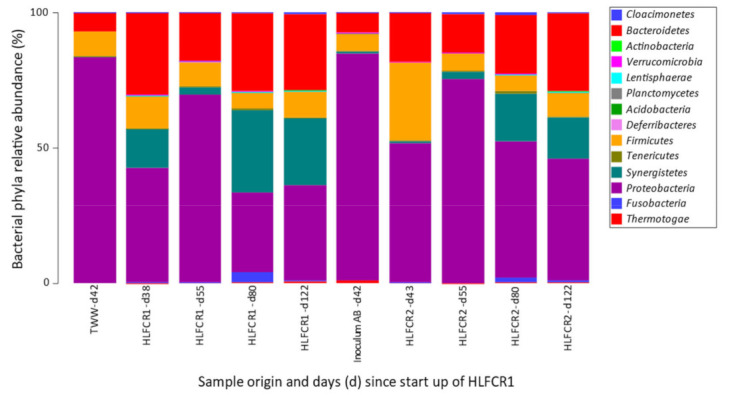
Bar graph of the phyla identified using 16S rRNA amplicon sequencing.

*Proteobacteria* were the most abundant phyla in the TWW and inoculum AB (84% RA in each). *Firmicutes* (7–8%) and *Bacteriodetes* (7–9%) were also relatively well represented phyla in these samples (Figure 6). As alluded to in Section 3.1, Inoculum AB was pre-acclimated in batch 2 TWW, which explains the similarity and confirms the results shown in the nMDS plots (Figure 3), that the community composition in Inoculum AB was strongly influenced by the SRB communities endogenous in the TWW. In the HLFCRs, there was a notable selection of *Synergistetes* (from <1% in TWW and Inoculum AB to 15–30% in the HLFCRs by days 80 and 122), and *Bacteroidetes* (21–29% RA by days 80 and 122).

In terms of bacterial genera in HLFCR1 and HFLCR2, notable respective increases in the RA of *Dethiosulfovibrio* (14→29% and <1→12%), *Petrimonas* (21→24% and 17→26%) and *Desulfomicrobium* (8→9% and <1→25%) were found during the course of the experiment. *Petrimonas sulfuriphila* (type strain BN3^T^) and *Petrimonas mucosa* (type strain ING2-E5A^T^ = DSM 28695^T^ = CECT 8611^T^) were first described in 2005 [26] and 2016 [27], respectively, and the genus has recently been reclassified into the family *Dysgonomonadaceae* [28]. *Petrimonas* spp., like all members of the *Dysgonomonadaceae* family, are capable of fermenting a range of complex proteins and carbohydrates to CH_3_COO^−^ and CO_2_ [22,27]. Both species of *Petrimonas* are strictly anaerobic chemoorganotrophs commonly found in mesophilic anaerobic digesters [27,28], where increased abundance of *D. mucosa* has been correlated with unstable reactor performance. It appears that the metabolic versatility of the genus provides it with superior stress resistance, allowing it to out-compete other genera [28]. Of note in this study is that *P. sulfuriphila* can use S^0^ as a terminal electron acceptor, reducing it to H_2_S, and growth is stimulated by the presence of S^0^ [26]. The protein-rich TWW and the presence of S^0^ from HS^−^ oxidation would have provided an ideal competitive environment for proliferation of *P. sulfuriphila* in the HLFCRs. Indeed, strains of *Petrimonas* and *Desulfovibrio* have recently been found co-dominating in a SO_4_^2−^ and aromatic hydrocarbon contaminated environment and hypothesized synergistic metabolic interactions between the two [29]. 

Another strongly selected genus, *Dethiosulfovibrio*, currently includes five known species that all share metabolic similarities with *Petrimonas. Dethiosulfovibrio peptidovorans* was described in 1997 [30] followed by *Dethiosulfovibrio russensis, Dethiosulfovibrio marinus*, *Dethiosulfovibrio acidaminovorans* [31] and *Dethiosulfovibrio salsuginus* [32]. *D. marinus*, *D. acidaminovorans* and *D. russensis* were isolated from sulfur mats, similar in nature to the FSB, where they were initially present in filamentous forms [31]. All species of *Dethiosulfovibrio* are strict anaerobes capable of utilizing proteins, peptides and amino acids as energy sources, but unlike *Petrimonas* cannot utilize sugars, although some can ferment organic acids [31,32,33]. All species are slight halophiles and not only reduce S^0^ (like *Petrimonas*) but can also reduce thiosulfate (S_2_O_3_^2−^) to H_2_S [30,33,34]. Like *Petrimonas* spp., the metabolic capabilities of *Dethiosulfovibrio* spp. clearly explains their competitive selection in the HLFCRs. In HLFCRs, the selection and dominance of these S^0^ reducing genera is not desirable as it counters the formation of S^0^ for recovery and removal of HS^−^ as a pre-treatment measure for AD. Their proliferation is most likely a consequence of slower or incomplete biofilm formation which resulted in increased O_2_ penetration. This led to partial SO in the upper layer of the bulk liquid, rather than exclusively within the biofilm. The existence of colloidal S in the anaerobic liquid bulk volume would have provided a source of S for the functional bacteria. 

Another noteworthy finding was that *Arcobacter*, detected in high RA in this study, has been shown to dominate in biofilms in highly sulfidic saline environments [35], and has been found environmental FSBs similar to those encountered in the HLFCRs. 

**Figure 7:**
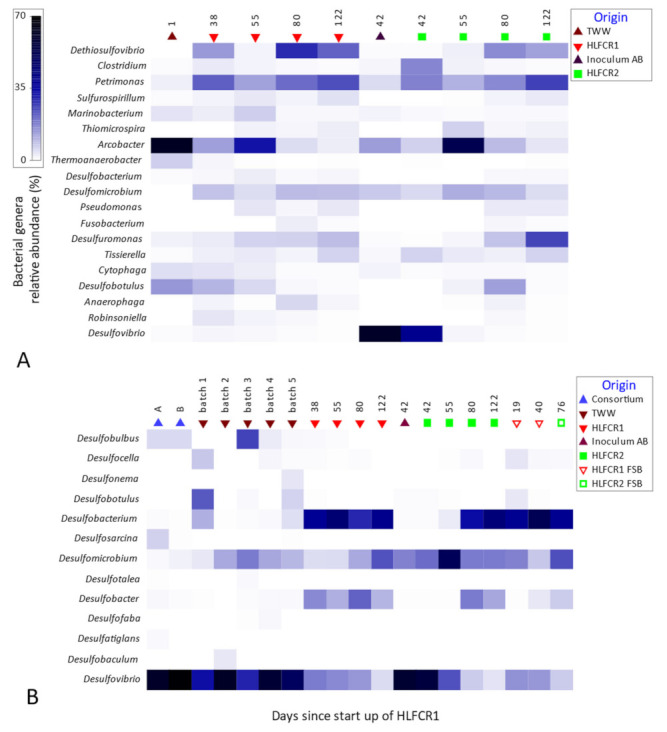
Shadeplot of the most abundant bacterial (**A**) and sulfite reducing (**B**) bacterial genera determined using 16S rRNA gene and *dsrB* primer sets, respectively.

For the remainder, the RA of *Clostridium* (2→<1% and 17→<1%) and *Arcobacter* (13→2% and 13→4%) decreased in HLFCR1 and HLFCR2, respectively, while *Desulfobotulus* decreased in HLFCR1 (10→<1%), and other genera such as *Desulfuromonas* remained relatively stable in both HLFCRs (Figure 7A). *Desulfovibrio* was found in high RA in the inoculum (61%) and during start-up of HLFR2 (39%) but decreased to <1% by the end of the study. In general, although all the most abundant genera were present in both batch 2 TWW and Inoculum AB, the RA profiles bore little resemblance to those in the HLFCRs at the end of the study (Figure 7A). This demonstrated a strong selective pressure exerted by the physicochemical mileu during operation of the HLFCRs. Notably, with the 16S rRNA primer set, many of the 19 most abundant genera have been associated with metabolism of S species. These include six dedicated SRB (*Dethiosulfovibrio*, *Desulfobacterium*, *Desulfomicrobium*, *Desulfuromonas*, *Desulfobotulus*, *Desulfovibrio*) and two other genera known to contain SRB species, namely, *Clostridium* [36] and *Tissierella* [37]. *Thiomicrospira* [38], *Arcobater* [35], some species of *Thermoanaerobacter* (e.g., *T. sulfurigignens* [39]), and *Pseudomonas* [40] are known oxidizers of S species. 

Examination of the results obtained with the SRB specific primer set (Figure 7B) showed that *Desulfovibrio* was present in high RA in the enriched consortia (A = 79%, B = 89%), the batches of TWW (38–79%) and the inoculum (76%) but decreased notably in HLFCR1 (23→6%) and HLFCR2 (31→9%) during the course of the experiment. This decrease, together with the increase in *Desulfomicrobium* and similar trends being found with *Desulfobotulus* substantiated the results obtained using universal bacterial primers. However, failure of the latter to detect *Dethiosulfovibrio* and *Desulfuromonas* was notable and highlights the need to use more than one primer set when evaluating microbial community compositions because primer pairs shown to have good coverage in one environment may not amplify critical OTUs from other environments [41]. 

**Figure 8:**
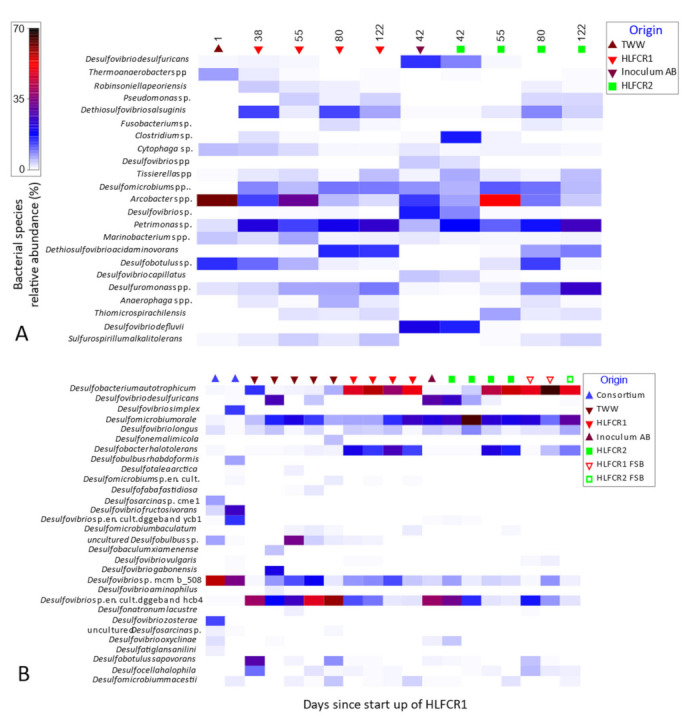
Shadeplot of the most abundant bacterial (**A**) and sulfite reducing (**B**) bacterial species determined using 16S rRNA gene and *dsrB* primer sets, respectively.

At species level, the temporal changes in the RA of the most abundant bacterial species in the TWW, inoculum AB and HLFCRs were assessed to ascertain which species were out-competed, preferentially selected or were resilient within the HLFCRs (Figure 8 and Table 3). *Desulfobacterium autotrophicum* and *Desulfobacter halotolerans* were strongly selected, and *Desulfomicrobium orale* remained resilient within the HLFCRs, reaching maxima of 57%, 27%, and 65% RA, respectively in the HLFCRs. 

*D. autotrophicum* is a complete organic substrate oxidizer, capable of heterotrophically degrading a variety of carbon sources, including acids, alcohols and long chain fatty acids [42,43]. It utilizes S_2_O_3_^2−^ and SO_4_^2−^ as electron acceptors for heterotrophic growth [43,44] and is capable of reducing SO_4_^2−^ at both high and low concentrations [45]. It is also able to grow chemolithoautotrophically by oxidizing H_2_ and CO_2_ [42,43], employing the Wood-Ljungdahl pathway to fix CO_2_ and completely oxidize acetyl-CoA to CO_2_ [42]. The metabolic versatility of this species makes it capable of adapting to changing environments. It can therefore play a pivotal functional role in HLFCRs treating TWW, which is inherently variable in nature. Furthermore, because *D. autotrophicum* is incapable of utilising S^0^ as an electron acceptor [44], it cannot interfere with formation of FSBs in HLFCRs. 

*D. halotolerans* is capable of growth in hypersaline environments containing up to 13% NaCl [46], a factor that allowed it to proliferate in the saline TWW. It has been shown to use ethanol and lactate as sources of organic carbon and, like *D*. *autotrophicum*, it is a complete CH_3_COO^−^ oxidizer [44,46]. *D. orale* has been found to oxidize lactate and pyruvate incompletely to CH_3_COO^−^ [47], and it was postulated that it occupied an important syntrophic metabolic niche in the HLFCRs. Of interest is that *D. orale* has previously been shown to be inhibited by HS^−^ [47] an assertion which appears to contradict the resilience of this species in the HLFCRs where concentrations of 400–500 mg/L HS^−^ were found. 

### 3.4. Dissimilatory Sulfite Reducing Gene Abundance and Sulfate Reduction Rates

The copy numbers of the *dsrB* gene were determined in the eluted DNA from centrifuged pellets of inoculum AB, batches of TWW, and the bulk liquid of the HLFCRs (Table 4). The concentration of DNA extracted from a specific sample volume has been used as a proxy to semi-quantitatively compare microbial abundance where other methods of biomass quantification, such as direct counting or gravimetric analysis are not possible [48,49,50,51,52]. In this case, the starting volume for DNA extraction from inoculum AB and the bulk liquid was 16 mL, while 4 mL was used for the TWW. Higher volumes of inoculum AB and bulk liquid were required in order to obtain sufficient material for extraction, assumed to be due to lower amounts of non-microbial particulates being present in these samples than in the TWW samples. In addition, eukaryotic DNA of animal origin would almost certainly have been present in the TWW, and it was postulated that this would skew the results to some extent. Due to these inconsistencies, it was not possible to make valid comparisons between the different sample types in terms of DNA concentration and functional gene abundance. Therefore, the discussion is separated into results obtained from the TWW and those obtained from the HLFCRs. 

Although TWW batch 2 exerted a significant influence on the SRB community structure in the HLFCRs, the lowest copy numbers (1–2 orders of magnitude) of the *dsrB* gene were found in this batch of TWW. 

In the HLFCRs, there was a notable increase in DNA concentration within each reactor over the course of the experiment, strongly suggesting an increase in microbial biomass. There was also a general increase in *dsrB* copy numbers and SRR after the start-up of HLCFR1 (day 12–38), which stabilised to some degree thereafter. In contrast, there was a decreasing trend in *dsrB* copy numbers as well as SRR in HLFCR2, corresponding to the decrease in SO_4_^2−^ concentration in the bulk liquid, suggesting that the substrate concentration impacts on the proliferation of the SRB. The competitive selection of S^0^ utilising *Dethiosulfovibrio* sp. and *Petrimonas* sp. is consistent with the reduced FSB formation from day 75 [14] as well as the lower SRR and *dsrB* copy numbers in HLFCR2. The proliferation of *Petrimonas* sp. also explains how the bulk concentration of HS^−^ in HLFCR2 (430 mg/L) remained similar to that in HLFCR1 (520 mg/L) despite receiving an influent with less SO_4_^2−^ (1000–1700 mg/L) than HLFCR1 (2400 mg/L). 

There appeared to be a moderate correlation (Pearson’s) between *dsrB* copy numbers and SRR in both bioreactors (r = 0.627), and in HLCFR1 on its own (r = 0.802), but sample numbers used in the statistical analysis were low (*n* = 8, and *n* = 5, respectively), and the results were not statistically significant (*p* > 0.05). 

## 4. Conclusions

The results of the study showed that the endogenous functional bacteria in TWW were more resilient in the HLFCRs than those harvested from saline estuaries. This indicates that bioaugmentation of HLFCRs during start-up is not necessary. The non-augmented HLFCR achieved a SRR of 600 mg/L.day and near complete SO, similar to results obtained using active methods [8]. To ensure that a diverse community of SR and SO bacteria are available for competitive selection, it is recommended that a number of batches of TWW from a tannery wastewater treatment plant are used to start-up biological systems for S removal from TWW. 

The proliferation of *Dethiosulfovibrio* sp. and *Petrimonas* sp. are consistent with the slow or incomplete biofilm formation and offer a plausible explanation for declining S^0^ recovery. This would not have been evident if only chemical results were available *viz*. if the systems were treated as ‘black boxes.’ The findings provide a key to improving the performance of the HLFCRs, either by promoting more rapid and complete biofilm formation or by determining means of inhibiting the growth of *Dethiosulfovibrio* sp. and *Petrimonas* sp. 

The HLFCRs were able to pre-treat TWW sufficiently to reduce S-related inhibition of downstream AD. In terms of the bacterial communities, temporal community stabilization coincided with more stable system performance.

The microbial succession in the HLFCRs showed a notable increase in the RA of species capable of utilizing proteinaceous substrates and reducing S^0^ (*Dethiosulfovibrio* sp. and *Petrimonas* sp.) and reducing SO_4_^2−^ (*D. autotrophicum* and *D. halotolerans*). The TWW and AB inoculum contained little S^0^, but this accumulated in the FSB and as colloidal sulfur in the HLFCRs. Biofilm formation was slower than in previous studies using simple, synthetic feed solutions and reduction of biofilm S^0^ could reduce S^0^ recovery and increase the risk of undesirable HS^−^ concentrations in the effluent. Future studies should focus on varying operational conditions to promote more rapid biofilm formation and discourage the selection of S^o^ reducing bacteria. 

## Figures and Tables

**Table 1:** Characteristics of tannery wastewater used in this study (*n* = 5 batches) adapted from [14].

Parameter	H-TOC TWW		L-TOC TWW	
	Average	SD	Average	SD
pH	10.24	1.6	7.81	0.52
EC (mS/cm)	32.01	2.2	31.5	1.59
TOC (mg/L)	6116	1875	886	253
COD (mg/L)	28,169	3665	4968	2940
BOD (mg/L)	6200	812	1539	520
VOA_t_ (mg/L AAE)	2920	718	1041	647
Protein (mg/L)	2875	1024	310.6	83.9
TN (mg/L)	1258	215	679	138
TAN (mg/L NH_3_-N)	301	286	350	235
NO_3_^−^ (mg/L)	70.8	25.0	40.5	23.6
NO_2_^−^ (mg/L)	5.1	5.2	2.35	2.63
PO_4_^3−^ mg/L	0	0	1.21	1.96
SO_4_^2−^ (mg/L)	1951	574	3687	383
HS^−^ (mg/L)	699	114	83	76
Cl (mg/L)	7744	460	7713	325
TS (g/L)	36.1	5.8	17.96	3.23
TVS (g/L)	13.1	3.3	1.93	0.47
K (mg/L)	95.7	31.1	100.3	19.5
Na (mg/L)	6412	571	6225	276
Fe (mg/L)	0.11	0.08	0.19	0.12
Ca (mg/L)	692	482	230.9	58.5
Mg (mg/L)	120	138	220.7	27.6
Mn (mg/L)	0.50	0.41	15.14	6.92
Zn (mg/L)	0.50	0.33	0.19	0.12
Cr (mg/L)	0.09	0.05	0.20	0.08
Alk (mg/L CaCO_3_)	3256	907	1999	385
COD:SO_4_^2−^	15.4	3.5	1.4	0.76
TVS:TS	0.36	0.03	0.11	0.02
BOD:COD	0.23	0.05	0.37	0.14
C:N	5.1	2.2	1.3	0.21
VOA:Alk	0.94	0.27	0.48	0.24
COD:TVS	2.2	0.52	2.5	0.93

TOC = total organic carbon; H-TOC = high TOC; L-TOC = low TOC; TWW = tannery wastewater; SD = standard deviation from the mean; EC = electrical conductivity; mS = milliSiemens; mV = millivolts; COD = chemical oxygen demand; BOD = biological oxygen demand; VOA_t_ = total volatile organic acids; AAE = acetic acid equivalents; TN = total nitrogen; TAN = total ammonia nitrogen; NH_3_-N = ammonia nitrogen; NO_3_^−^ = nitrate; NO_2_^−^ = nitrite; PO_4_^3−^ = phosphate; SO_4_^2−^ = sulfate; HS^−^ = hydrogen sulfide; TVS = total volatile solids; TS = total solids; Alk = alkalinity; CaCO_3_ = as calcium carbonate; C:N = carbon to nitrogen ratio.

**Table 2:** ANOSIM significant R values (*dsrB* amplicon Global R = 0.435 ***).

	HLFCR2 (*n* = 4)	HLFCR1 (*n* = 4)	TWW (*n* = 5)
HLFCR1 (*n* = 4)	NS	-	-
TWW (*n* = 5)	NS	0.75 *	-
Consortia (*n* = 5)	0.956 **	1 **	0.988 **

Significance levels: <0.05 * ≥ 0.01 > **0.005 ≥ *** NS = not significant.

**Table 3:** Semi quantitative analysis of abundance of different *dsrB* containing bacterial species.

	Inoc. AB	TWW *	HLFCR *	Comments
**Selected species**				
*Desulfobacterium autotrophicum*	+	++	++++	-
*Desulfobacter halotolerans*	+	+	+++	-
*Desulfomicrobium macestii*	+	+ to ++	+ to ++	Temporal increase in RA
**Species outcompeted**				
*Desulfobacterium desulfuricans*	+++	++	+	-
*Desulfovibrio longus*	++	++	++ to +	Temporal decrease in RA
*Uncultured Desulfobulbus* sp.	+	+++	+	Temporal decrease in HLFCR1
*Desulfobaculum xiamenense*	ND	+/++	ND	TWW batches 2 & 5 only
*Desulfovibrio gabonensis*	+	+/+++	+	Only +++ in TWW batch 2
*Desulfobacterium sapovorans*	+	+/++	+	Temporal decrease in RA
*Desulfocella halophila*	ND	+/++	+	Temporal decrease in RA
*Desulfovibrio* sp. mcm b_508	++	++/+++	++ to +	Slow temporal decrease in RA
*Desulfovibrio enrichment culture dgge band hcb4*	+++	+++/++++	++ to +	Temporal decrease in RA
Resilient species				
*Desulfomicrobium orale*	+++	+++	+++	-

* Range in different batches (1–5) of TWW or HLFCRs. + scanty, ++ moderate, +++ abundant, ++++ highly abundant, RA = relative abundance.

**Table 4:** Comparison of functional (*dsrB*) gene abundance and sulfate reduction rates.

Sample	Batch or Time (Days)	DNA Conc. (ng/µL)	*dsrB* Copy Numbers (Number/ng DNA)	SR Rate (mg/L·day)
TWW	Batch 1	25	2.11 × 10^5^	NA
	Batch 2	3	8.62 × 10^3^	NA
	Batch 3	6	2.10 × 10^4^	NA
	Batch 4	5	1.24 × 10^4^	NA
	Batch 5	18	1.56 × 10^4^	NA
HLFCR1	12	25	2.11 × 10^5^	198
	38	96	3.64 × 10^5^	674
	55	87	4.49 × 10^5^	537
	80	64	3.85 × 10^5^	643
	112	186	3.65 × 10^5^	568
Inoculum AB	42	237	9.25 × 10^5^	NA
HLFCR2	42	137	6.31 × 10^5^	NA
	55	184	4.11 × 10^5^	439
	80	113	3.46 × 10^5^	182
	123	230	1.49 × 10^5^	281

NA = not applicable, SR = sulfate reduction.

## Data Availability

Raw sequence data obtained in this study has been deposited in the National Center for Biotechnology Information Database under BioProject PRJNA89991.

## Abbreviations

AM = acetoclastic/acetotrophic methanogens; FSB = floating sulfur biofilm; HLFCR = hybrid linear flow channel reactor; HM = hydrogenotrophic methanogens; SAOB = syntrophic acetate oxidizing bacteria; SO = sulfide oxidation; SR = sulfate/sulfide reduction; SRB = sulfate reducing bacteria; SRR = sulfate reduction rate.
